# Trend and Factors Associated with Medical–Surgical Complications in Patients Discharged from Leprosy Multidrug Therapy at the Specialized Regional Hospital in Macenta, Guinea, from 2012 to 2021

**DOI:** 10.3390/tropicalmed9120290

**Published:** 2024-11-28

**Authors:** Jean Hébélamou, Fassou Mathias Grovogui, Hawa Manet, Lavilé Povogui, Ismael Béavogui, Karifa Kourouma, Abdoulaye Sow, Alexandre Delamou

**Affiliations:** 1 Centre Hospitalier Régional Spécialisé (CHRS) de Macenta, Macenta GGRH+69Q, Guinea; povoguilavile@gmail.com (L.P.); ismaelbeavogui799@gmail.com (I.B.); 2 Centre National de Formation et de Recherche en Santé Rurale de Mafèrinyah, Forécariah GPW7+V9G, Guinea; mgrovogui@cea-pcmt.org (F.M.G.); hawa@maferinyah.org (H.M.); kkourouma@maferinyah.org (K.K.); adelamou@cea-pcmt.org (A.D.); 3 Africa Center of Excellence for the Prevention and Control of Communicable Diseases (CEA-PCMT), University Gamal Abdel Nasser of Conakry, Conakry BP 1147, Guinea; drsowab@msn.com

**Keywords:** leprosy, medical–surgical complications, CHRS Macenta, Guinea

## Abstract

This study analyzed the trend and factors associated with medical–surgical complications in patients discharged from leprosy multidrug therapy at the Centre Hospitalier Régional Spécialisé (CHRS), in Macenta, Republic of Guinea. This was a retro 2012 (*n* = 54) and 2013 (*n* = 35) and then a slight decrease between 2014 (*n* = 34) and 2017 (*n* = 26). From 2019 (*n* = 18) to 2021 (*n* = 1), a significant d spective study using routine secondary data from 2012 to 2021. The most represented age group ranged from 25 to 59 years (73.8%), with a male predominance of 72.6%. Farmers represented 60.7% of the patients, 74.5% of the patients had plantar wounds, and 48.8% resided in the N’zerekore region. A trend analysis showed an overall significant decrease in the number of patients with complications between ecline was found. In the patients with leprosy reactions, there was a reduction in numbers from 48 in 2012 to 2 in 2014, with a predominance in men. There were significant associations between region, plantar perforation disease (*p* = 0.013), and physical disability (*p* = 0.029) and between year and leprosy reaction after the cure (*p* < 0.001). In summary, there was a high proportion of patients with plantar ulcers, which predominantly affected farmers, and a significant proportion with leprosy reactions and physical disabilities. Community awareness around leprosy and capacity building of the providers in terms of appropriate management may contribute to improving patients’ quality of life.

## 1. Introduction

Leprosy is a chronic bacterial infection caused by Mycobacterium leprae and Mycobacterium lepromatosis, which preferentially affect the skin, nerves, limbs, and eyes. It is highly contagious but only causes real illness in a few infected people, with serious consequences not only for their health but also for their socio-economic situation. Medical–surgical complications are due to disabilities caused by neural peripheral leprosy. Sequelae, such as plantar ulcers, can occur several years after recovery and sometimes require amputation of the limb [1,2]. 

According to the World Health Organization (WHO), 129,192 new cases of leprosy were recorded in 161 countries worldwide in 2021. Africa recorded 14,859 new cases, representing a prevalence of 0.19 in 10,000 people [3].

The prevalence of leprosy-related disability varies from one setting to another, reflecting the capacity of countries to manage leprosy cases early. In a published study of 896 new leprosy cases detected between 2015 and 2019 in Chad, Kabo et al. reported grade 2 disability in 20.4% of patients [4]. In Ethiopia, Masresha et al. reported a prevalence of leprosy-related disability in 34.6% of their patients during treatment [5]. Similarly, Mustapha et al., in 2019, reported a prevalence of plantar ulceration in 51.1% of the patients managed in referral hospitals in northern Nigeria [6]. There is no recent study on the national prevalence of leprosy-related disability in Guinea. According to the National Program for the Control of Neglected Tropical Diseases (NPCNTD), the prevalence was estimated at 9% in 2008. In a study conducted in 2016 by Keita M. et al., 60 cases of leprosy-related plantar-perforating injuries were recorded in Conakry’s leprosy management centers [7].

The main factor associated with these disabilities is a delay in diagnosis and management [8]. Other studies have also shown that gender and socio-economic status are associated with the occurrence of disability [8].

Leprosy control in Guinea is based on several strategies, including early case detection and management, community involvement, and prevention of complications or disabilities in all new cases. These strategies have reduced the prevalence of the disease from 12 to less than 1 case per 10,000 inhabitants and the prevalence of complications from 16.7% to 8% between 1990 and 2020 [8]. However, there are still hyper-endemic areas in some districts of the country, and these resulted in the detection of 250 new leprosy cases in 2017 [8]. According to the WHO, many patients who are not disabled before admission will develop disability during or after treatment. The NPCNTD is a vertical program that provides support for the management of leprosy and its complications through dedicated sites, including the CHRS in Macenta.

Little is known to date about the progress in the number of medical and surgical complications in patients who have been discharged from leprosy multidrug therapy in Guinea and the patient characteristics that may determine these complications. The aim of this study, therefore, was to analyze the trend in medical–surgical complications and their possible determinants among patients discharged from leprosy multidrug therapy at the CHRS in Macenta, Guinea, between 2012 and 2021. The specific objectives were the following:

- Describe the socio-demographic and clinical characteristics;
- Determine the trend in medical and surgical complications;
- Analyze the determinants of medical–surgical complications.

The results of this study should help the program develop strategies to prevent complications, improve the management of patients, and, ultimately, restore a better quality of life.

## 2. Materials and Methods

### 2.1. Study Design

This was an analytical cross-sectional study that used secondary data on the management of leprosy patients treated at the CHRS in Macenta for medical and surgical complications between January 2012 and December 2021.

### 2.2. Study Setting

#### 2.2.1. General Setting

The Republic of Guinea is a West African country with an estimated population of about 13,132,792 inhabitants in 2022. Approximately 44% of the population live below the national poverty line, and 40% of adults cannot read and write.

The country is divided into eight administrative regions and Conakry, the capital city. The health system is tiered into a primary level (1640 health posts and 407 health centers), a secondary level (26 district hospitals, 8 medical community centers), and a tertiary level (3 national hospitals) [9]. Leprosy control in Guinea is organized and piloted by the NPCNTD. It is a vertical program with care sites, including the CHRS in Macenta.

#### 2.2.2. Specific Setting

The CHRS in Macenta was this study’s setting. It has a capacity of 126 hospital beds and 64 staff members. It is integrated into the local and regional health systems and serves as a regional reference center for leprosy, tuberculosis, HIV/AIDS, and hepatitis B. It also takes care of patients from other regions of Guinea, notably Faranah and Kankan. The management of NTD cases at the CHRS in Macenta had historically been ensured by the Mission Philafricaine (MPA), starting in 1983, before the withdrawal of funding in 2018. The MPA is a Swiss NGO that provided funding for prevention, disability care, and the physical and socio-economic rehabilitation of former leprosy patients.

### 2.3. Study Population and Period

The study population included all patients who attended the CHRS in Macenta for medical and surgical complications after recovery from leprosy between 1 January 2012 and 31 December 2021.

### 2.4. Data Collection

Data were extracted from the hospital registers. A data extraction form was developed to collect variables on the individual and clinical characteristics of the patients, including the types of complications which occurred after leprosy recovery. This form was developed in Microsoft Office Excel and served as a data entry form. Data were collected from 1 July to 31 August 2022 by the principal investigator and the data manager of the CHRS in Macenta.

### 2.5. Study Variables

#### 2.5.1. Dependent Variable

Our variable of interest was the presentation or manifestation of a medical–surgical complication in individuals discharged from leprosy multidrug therapy. The complications considered were post-cure leprosy reactions, plantar wounds, palmar wounds, leg wounds, peripheral neuropathy, and lagophthalmos. Since each patient could have more than one complication at a time, each of the above complications was coded 0 if not present in the patient and 1 if present.

#### 2.5.2. Independent Variables

The independent variables included individual patient characteristics such as age, gender, place of residence, occupation, and year of admission. For the purpose of data analysis, some of the variables were re-coded. The patient’s age was re-coded into three groups (15–24; 25–59; 60 or more), according to sex (male and female), region of origin (N’zerekore, Kankan, Faranah, and others, e.g., Mamou, Kindia, Conakry, Liberia, and Mali), occupation (farmer, housewife, and other, e.g., workman/workwoman, pupil, student, and civil servant).

### 2.6. Data Management and Analysis

The data were first processed using Microsoft Office Excel and then exported to Stata version 16 (Stata Corporation, College Station, TX, USA) for analysis. The qualitative variables were summarized as proportions, and the quantitative variables were described as medians with interquartile ranges (IQRs). The overall trend in medical–surgical complications was analyzed alongside specific trends in relation to the sex of the participants. The relationship between each of the individual patient characteristics and the occurrence of medical–surgical complications after leprosy recovery was analyzed using Pearson’s chi-square. The levels of significance were set at 5% (*p* < 0.05).

### 2.7. Ethics Considerations

The study protocol was approved by the National Health Research Ethics Committee of Guinea (number L-080-CNERS-22).

## 3. Results

### 3.1. Socio-Demographic and Clinical Characteristics of Patients Discharged from Leprosy Multidrug Therapy

The socio-demographic and clinical characteristics of the study participants are presented in Table 1.

Overall, 252 people attended the CHRS in Macenta for medical and surgical complications during the ten-year study period. The median age of the participants was 45 years, with an IQR of 32 to 55 years. More than three out of five people were men (73%) and farmers (61%). The participants were mainly from the N’zerekore (49%) and Kankan (24%) regions. The main reasons for hospitalization were foot sores (75%), followed by leprosy reactions (31%) and physical disabilities (11%). The median length of hospital stay was 78 days, with an IQR ranging from 57 to 114 days.

### 3.2. Trends in Medical and Surgical Complications in Patients Discharged from Leprosy Multidrug Therapy

**Figure 1:**
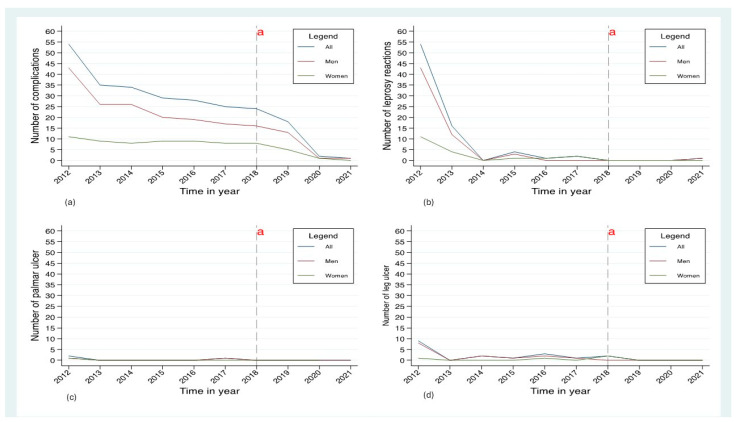
Trends in medical and surgical complications (overall and by gender) in patients discharged from leprosy multidrug therapy at the CHRS in Macenta, Guinea, between 2012 and 2021. (**a**) Overall reduction in complication rates between 2012 and 2021; (**b**) Decrease in cases of leprosy reactions between 2012 and 2014; (**c**,**d**) Low incidence of palmar and leg ulcers. ^a^ End of free care for leprosy and its complications at the CHRS in Macenta.

The trends in the medical and surgical complications among the patients cured of leprosy at the CHRS in Macenta between 2012 and 2021 are shown in Figure 1. The trend analysis shows an overall significant decrease in the number of cases of complications between 2012 (54 cases) and 2013 (35 cases) and then a slight decrease between 2014 (34 cases) and 2017 (26 cases). From 2019 (18 cases) to 2021 (1 case), a significant decline was found (see Figure 1a). The trend in male patients mirrored the overall trend, while, in female patients, there was little difference between the years, apart from a decline from 2018 to 2021 (Figure 1a). Among the patients with a leprosy reaction, there was a marked reduction from 2012 to 2014, and this was particularly notable in male patients (Figure 1b). From 2014 to 2018, the situation remained stable until 2018, when there were no cases (Figure 1b). From 2012 to 2018, the number of cases of palmar ulcers remained relatively low (Figure 1c). Similarly, there was a decrease in patients with leg ulcers from 2012 to 2013, after which the numbers remained stable until 2021 (Figure 1d). The number of patients with plantar ulcers overall gradually declined over the ten-year period, with the trends being the same in male patients and less pronounced in female ones (Figure 1a). Over the ten-year period, there was a small number of patients with physical disabilities, lagophthalmos, and peripheral neuropathy (neuritis). 

### 3.3. Determinants of Medical and Surgical Complications 

The associations between socio-demographic characteristics and medical–surgical complications in patients discharged from leprosy multidrug therapy at the CHRS in Macenta, Guinea, from 2012 to 2021 are shown in Table 2a,b. The key findings were as follows: significant associations between region and plantar perforation disease (*p* = 0.013) and region and physical disability (*p* = 0.029); and a significant association between year and leprosy reaction after the cure (*p* < 0.001). 

## 4. Discussion

This study, which spanned a period of 10 years, is the first of its kind in the forest region of southern Guinea, as previous studies focused on the complications of leprosy as the disease progresses. Our study showed a high frequency of plantar ulcers among the participants (73%). This is because the sole of the foot supports the whole body during walking and prolonged standing, required in some agricultural activities, and the plantar area is, therefore, subject to a huge amount of pressure. In addition, insensitivity due to peripheral neuropathy exposes the area to plantar ulceration. Farmers were the most affected, unlike housewives. In their study, Keita et al. reported a high prevalence of foot ulcers, with the predisposing factors being foot deformity and local sensitivity disorders [7]. Our results are similar to those found by Gidado M et al. in northern Nigeria, who showed that half of the patients discharged from leprosy multidrug therapy had plantar ulcers, with a predominance in men [6]. The same study also showed that farmers were predominantly affected. This demonstrates the importance of awareness sessions among leprosy patients to ensure the protection of their feet by wearing closed shoes and the regular observation of the feet to look for micro-lesions that could become infected if not properly managed.

Our data also showed a considerable proportion of leprosy reaction cases during the period from 2012 to 2014. A plausible explanation is that this complication can occur during the course of the disease and even after recovery due to the complex interactions that occur between the host immune system and Mycobacterium lepromatosis. In our study, no details were provided on the different types of leprosy reactions. However, in a study conducted in 2022 in Brazil, Goulart I.M.B. et al. reported a case of leprosy reaction in a 57-year-old man after multidrug therapy that manifested as arthritis, erythematous nodules, and tibial nerve neuritis four months after recovery [10]. Sardana K. et al., in another study conducted in India in 2020, found that cases of leprosy reactions were rare. Only one case of leprosy reaction in the form of erythema multiforme (EM) was found 1 year after the completion of multidrug therapy; no triggering factors could be identified [11]. It is, therefore, important to continue to surveil cured leprosy patients and plan strategies for the management of complications arising after the end of a specific treatment.

Another finding of our study was that just over 10% of the participants had a physical disability. This could be explained by the late access to health care structures, which favors neurological damage, leading to limb deformation or the chronic development of palmoplantar ulcers, leading to amputations. The number of cases of physical disability found in our study varied from two to six cases per year. These results are similar to those found by Carlos Dornels F.S. et al. in Brazil in 2019, who found a number of cases of complications with physical disability ranging from 5 to 10 cases per year [11]. In this regard, it is important to organize community awareness sessions for the early detection of disability and strengthen the capacity of health care providers for early diagnosis and case management.

Our trend analysis showed an overall decrease in the number of cases of complications from 2012 (54 cases) to 2021 (1 case). These results are probably due to the decentralization of patient management in the various centers around the country for the prevention and management of complications. From 2014 onwards, the number of cases of complications remained almost stable until 2018. This stability can be explained by the outbreak of the Ebola virus disease, which affected the Guinean health system, leading to the underutilization of care services. From 2018 to 2019, there was an overall gradual decline in the number of cases. This period corresponds to the transitional phase proposed by the Mission Philafricaine (MPA) to stop free treatment for new and old leprosy patients. After 2019, the drop was significant until 2021, when only one complication case was reported. This drop in the number of cases after 2019 could be explained by the coronavirus pandemic. Data on the development of complications in patients discharged from leprosy multidrug therapy are scarce in the literature, and comparisons with our study are, therefore, difficult. 

The main limitation of this study is that it was a retrospective study and did not allow for the determination of certain independent variables such as socio-economic level and educational level. There was also the risk of missing data.

To determine the leprosy treatment, we used the following classic scheme, which has been in place since 1984:

1. Pauci bacillary leprosy: if the number of spots is less than five, the 6-month treatment schedule is used.
2. Multibacillary leprosy: if the number of spots is greater than five, the 12-month treatment schedule is used.

## 5. Conclusions

Our study revealed a high proportion of patients with plantar ulcers, which mainly affected farmers, and a substantial proportion of leprosy reactions and physical disabilities. In addition, a small proportion of other complications, including neuritis, leg ulcers, and lagophthalmos, were found. Community awareness of leprosy, producing sandals with flexible soles, and strengthening the capacity building of health care workers to allow for earlier diagnoses and appropriate management should contribute to improving the quality of life of the patients.

## Figures and Tables

**Table 1:** Socio-demographic and clinical characteristics of patients discharged from leprosy multidrug therapy at the CHRS in Macenta, Guinea, from 2012 to 2021 (*n* = 252).

Characteristics	Number	Percentage
Median age in years	55 (IQR = 32–55)	
Median length of hospital stay in days	78 (IQR = 57–114)	
Age group in years		
15–24	17	6.7
25–59	186	73.9
60–82	49	19.4
Gender		
Male	183	72.6
Female	69	27.4
Occupation		
Farmer	153	60.7
Housewife	72	28.5
Pupil/Student/Public Servant	5	2.0
Workman/woman	11	4.4
Seller	11	4.4
Clinical manifestations		
Plantar wound	187	74.5
Plantar perforation disease	184	73.0
Leprosy reaction after healing	78	31.0
Physical disability	29	11.5
Leg wound	18	7.1
Neuritis (peripheral neuropathy)	4	1.6
Palmar wound	3	1.2
Lagophthalmos	2	0.8
Region of origin		
N’zerekore	123	48.8
Kankan	60	23.8
Faranah	50	19.8
Others ^1^	19	7.6

Two (2) participants were under the age of 15; because of their small number, we have associated them with the age group of 15–24. No new antibiotic treatment (leprosy multidrug therapy) was given to previously treated patients with a leprosy reaction. ^1^ Bamako, Mali = 2 cases; Liberia = 1 case; Mamou = 2; Kindia = 6; and Conakry = 8.

**Table 2:** (**a**): Socio-demographic characteristics and medical–surgical complications in patients cured of leprosy at the CHRS in Macenta, Guinea, from 2012 to 2021 (*n* = 252). (**b**): Socio-demographic characteristics and medical–surgical complications in patients discharged from leprosy multidrug therapy at the CHRS in Macenta, Guinea, from 2012 to 2021 (*n* = 252).

(a)									
Characteristics	Number	Leprosy Reaction after Cure		Palmar Wound		Leg Wound		Plantar Perforation Disease	
		Yes (%)	*p*-Value	Yes (%)	*p*-Value	Yes (%)	*p*-Value	Yes (%)	*p*-Value
Age group, years									
15–24	17	29.4	0.987	0.0	0.584	5.9	0.940	76.5	0.777
25–59	186	31.2		1.6		7.0		74.7	
60–82	49	30.6		0.0		8.2		79.6	
Gender									
Male	183	32.2	0.471	1.1	0.816	7.7	0.611	76.0	0.922
Female	69	27.5		1.4		5.8		75.4	
Profession									
Farmer	153	29.4	0.120	2.0	0.374			75.8	0.746
Housewife	72	27.8		0.0		7.2	0.633	77.8	
Other ^1^	27	48.1		0.0		5.6		70.4	
Region									
N’zerekore	123	29.3	0.642	0.8	0.345	11.4	0.055	74.8	0.013
Faranah	50	34.0		0.0		6.0		84.0	
Kankan	60	28.3		3.3		1.7		80.0	
Other ^2^	19	42.1		0.0		0.0		47.4	
Year									
2012	54	100.0	0.001	3.7	0.686	16.7	0.147	66.7	0.248
2013	35	45.7		0.0		0.0		85.7	
2014	34	0.0		0.0		5.9		79.4	
2015	29	13.8		0.0		3.4		62.1	
2016	28	3.6		0.0		10.7		71.4	
2017	26	7.7		3.8		3.8		76.9	
2018	25	0.0		0.0		8.0		84.0	
2019	18	0.0		0.0		0.0		88.9	
2020	2	0.0		0.0		0.0		100.0	
2021	1	100.0		0.0		0.0		100.0	
**(b)**									
**Characteristics**	**Number**	**Physical Disability**		**Lagophthalmos**		**Neuritis**		**Outcome**	
		**Yes (%)**	***p*-Value**	**Yes (%)**	***p*-Value**	**Yes (%)**	***p*-Value**	**Yes (%)**	***p*-Value**
Age group, years									
15–24	17	5.9	0.751	0.0	0.533	0.0	0.336		
25–59	186	11.8		0.5		3.2		0.0	0.670
60–82	49	12.2		2.0		0.0		2.7	
								4.1	
Gender									
Male	183	13.1	0.193	0.5	0.471	1.6	0.209		
Female	69	7.2		1.4		4.3		2.7	0.943
								2.9	
Profession									
Farmer	153	13.7	0.330	0.0	0.080	2.0	0.414		
Housewife	72	6.9		2.8		4.2		2.0	0.613
Other ^1^	27	11.1		0.0		0.0		4.2	
								3.7	
Region									
Nzerekore	123	10.6	0.029	0.0	0.066	2.4	0.074		
Faranah	50	12.0		2.0		2.0		1.6	0.523
Kankan	60	6.7		0.0		0.0		2.0	
Other ^2^	19	31.6		5.3		10.5		5.0	
								5.3	
Year									
2012	54	13.0	0.901	1.9	0.907	0.0	0.405		
2013	35	8.6		0.0		2.9		7.4	0.617
2014	34	14.7		2.9		0.0		2.9	
2015	29	13.8		0.0		6.9		2.9	
2016	28	17.9		0.0		0.0		3.4	
2017	26	7.7		0.0		7.7		0.0	
2018	25	4.0		0.0		4.0		0.0	
2019	18	11.1		0.0		0.0		0.0	
2020	2	0.0		0.0		0.0		0.0	
2021	1	0.0		0.0		0.0		0.0	

^1^ Other: seller, pupil, student, farmer, and civil servant. ^2^ Other: Conakry, Mamou, Kindia, Vonzama (Libéria), and Bamako (Mali).

## Data Availability

The original contributions presented in the study are included in the article, further inquiries can be directed to the corresponding author/s.
